# Does Body Mass Index and Height Influence the Incident Risk of Ischemic Stroke in Newly Diagnosed Type 2 Diabetes Subjects?

**DOI:** 10.1155/2019/2591709

**Published:** 2019-01-22

**Authors:** Donghui Duan, Hui Li, Jiaying Xu, Liping Wong, Guodong Xu, Fanqian Kong, Sixuan Li, Qinghai Gong, Xiaohong Zhang, Jinshun Zhao, Lina Zhang, Guozhang Xu, Wenhua Xing, Liyuan Han

**Affiliations:** ^1^Department of Preventive Medicine, Zhejiang Provincial Key Laboratory of Pathophysiology, School of Medicine, Ningbo University, Ningbo, China; ^2^Institute of Non-Communicable Disease Control and Prevention, Ningbo Municipal Center for Disease Control and Prevention, Ningbo, China; ^3^Department of Epidemiology, School of Public Health and Tropical Medicine, Tulane University, New Orleans, USA; ^4^Department of Social Preventive Medicine, Faculty of Medicine, University of Malaya, 50603 Kuala Lumpur, Malaysia

## Abstract

**Objective:**

To estimate the incident risk of ischemic stroke (IS) in newly diagnosed type 2 diabetes (T2D) subjects according to different body mass index (BMI) and height categories.

**Methods:**

A total of 25,130 newly diagnosed T2D subjects were included in this study. All T2D subjects were enrolled consecutively from the Chronic Disease Surveillance System (CDSS) of Ningbo. Standardized incidence ratio (SIR) and its 95% confidence interval (95% CI) stratified by BMI categories and height quartiles were used to estimate the incident risk of IS in T2D subjects.

**Results:**

In total, 22,795 subjects completed the follow-up. Among them, 1268 newly diagnosed IS cases were identified, with 149,675 person-years. The SIRs of normal BMI (18.5–24.0 kg/m^2^), overweight (24.0–28.0 kg/m^2^), and obese (≥28.0 kg/m^2^) in overall subjects were 2.56 (95% CI 1.90–3.13), 2.13 (95% CI 1.90–3.13), and 1.87 (95% CI 1.29–2.43), respectively (*P*_trend_ < 0.01), comparing to the general population of Ningbo. For each 1 kg/m^2^ increment in BMI, the SIR was 0.948 (95% CI 0.903–0.999). For height quartiles, the SIRs of male subjects in quartile 1 (<160 cm), quartile 2 (161–165 cm), quartile 3 (165–170 cm), and quartile 4 (≥171 cm) were 2.27 (95% CI 1.99–2.56), 2.01 (95% CI 1.67–2.45), 1.37 (95% CI 1.05–1.68), and 0.91 (95% CI 0.40–1.32), respectively (*P*_trend_ < 0.01). While for female subjects, the SIRs in quartile 1 (<155 cm), quartile 2 (156–160 cm), quartile 3 (161–165 cm), and quartile 4 (≥166 cm) were 3.57 (95% CI 3.11–3.49), 2.96 (95% CI 2.61–3.31), 1.94 (95% CI 1.51–2.36), and 1.71 (95% CI 0.95–2.47), respectively (*P*_trend_ < 0.01).

**Conclusion:**

Compared to the general population of Ningbo, T2D subjects had a higher incident risk of IS. Furthermore, the IS incident risk was not only higher in newly diagnosed T2D subjects with normal BMI but also lower in taller newly diagnosed T2D subjects.

## 1. Introduction

Type 2 diabetes (T2D), a major public health burden, affects more than 370 million people around the world [1]. Accumulating evidence indicates that people with T2D have a threefold higher risk of stroke in some ethnicities [2–5]. Stroke, one of the leading causes of death in China, contributes to 1.6 million deaths annually [6–8]. Of which, about 80% of all patients suffer ischemic stroke (IS) [9].

Compelling evidence suggests a significant relationship between T2D and higher IS mortality [10]. Compared to overweight or obese subjects, those with normal weights were associated with higher IS mortality in both T2D subjects [11, 12] and the general population [4, 11–15]. Few studies have investigated the incident risk of IS in newly diagnosed T2D subjects based on different categories of BMI [16], especially in Asians. And epidemiology studies revealed an inverse association between height and risk of stroke in adults [17–19]. A large meta-analysis involving 121 prospective studies reported that each 0.065 cm increase in height was associated with 6% (95% CI 3–10%) decreased risk of IS [20].

Taken together, we hypothesized that different BMI categories or height quartiles would experience different incident risk of IS in newly diagnosed T2D subjects. Therefore, the aim of this study was to estimate the incident risk of IS in newly diagnosed T2D subjects based on different categories of BMI and height quartiles with standardized incidence ratio (SIR).

## 2. Material and Methods

### 2.1. Study Population

Ningbo, a coastal city with a population of over seven million in 2015, is an economic center in Zhejiang Province. All the newly diagnosed T2D subjects included in this study were obtained from the Chronic Disease Surveillance System (CDSS) of Ningbo. The CDSS was established based on 11 monitoring sites, which are fully representative of the whole of Ningbo. The system was founded in 2002, with the aim to monitor the epidemic of chronic diseases (diabetes, cardiovascular disease, and cancer) and their risk factors. Residents who have lived in Ningbo for more than five years are included in the CDSS [21]. A total of 25,130 subjects at baseline were included in this study in accordance with the following inclusion criteria: newly diagnosed T2D subjects; diagnosed between January 1, 2006, and December 31, 2007; had available health records in the CDSS; and without myocardial infarction, heart failure, or stroke at baseline. All the included subjects were followed once per year, and loss to follow-up was defined as could not be contacted after three reasonable efforts. This study was approved by the institutional review board of Ningbo Municipal Center for Disease Prevention and Control. All the subjects provided written informed consent.

### 2.2. Outcome

All the newly diagnosed T2D subjects at baseline were connected to stroke diagnosis records (diagnosed between January 1, 2008, and December 31, 2014) in the CDSS, through each subject's full name, personal ID, and gender. IS was diagnosed according to the Trial of Org 10172 in Acute Stroke Treatment (TOAST): a sudden onset of focal (or global) disturbance of cerebral function lasting > 24 h (unless interrupted by surgery or death) with no apparent nonvascular cause [22], with symptoms of large artery atherosclerosis, small artery occlusion, nonatherosclerotic vasculopathies, prothrombotic disorders, and cryptogenic cause [9, 22].

### 2.3. Demographic and Biochemical Measurements

Demographic and biochemical data were obtained from the medical records in the CDSS. Demographics included age, sex, weight, height, and education level. Biochemical measurements included fasting blood glucose (FBG), total cholesterol (TC), low-density lipoprotein cholesterol (LDL-C), high-density lipoprotein cholesterol (HDL-C), and glycated hemoglobin (HbA_1c_). Blood glucose levels were measured by modified hexokinase enzymatic method. TC and HDL-C were analyzed enzymatically using commercial reagents, and LDL-C levels were calculated using the Friedewald equation. HbA_1c_ was measured by ion-exchange HPLC on a Bio-Rad Variant II instrument [23].

### 2.4. BMI and Height Classification

BMI was calculated in kg/m^2^ according to each subject's height and weight. Weight was measured without shoes and in light clothing to the nearest 0.1 kg using a calibrated beam scale, and height of participants was measured without shoes to the nearest 0.2 cm using a portable stadiometer [24]. Subjects were divided into three groups: normal BMI group (18.5–24.0 kg/m^2^), overweight group (24.0–28.0 kg/m^2^), and obesity group (≥28.0 kg/m^2^) [25], in accordance with the criteria issued by the National Health and Family Planning Commission of China. Sensitivity analysis by applying the World Health Organization criteria [normal weight (18.5<25 kg/m^2^), overweight (25–29.9 kg/m^2^), and obese (≥30 kg/m^2^)] was also performed [26]. We also excluded the underweight subjects (BMI ≤ 18.5 kg/m^2^), as lower body weight tended to be coexistent with obesity-related metabolic disorders, which were more susceptible to stroke [11]. For height, subjects were divided into quartiles: ≤160 cm, 161–165 cm, 166–170 cm, and ≥171 cm for males and ≤155 cm, 156–160 cm, 161–165 cm, and ≥166 cm for females, respectively.

### 2.5. Statistical Analysis

Continuous variables were presented as mean ± SD and categorical variables as absolute and relative frequencies (percentage). Baseline characteristics were summarized based on BMI categories and height quartiles. Comparisons of demographic and clinical variables between males and females were performed using *t*-tests and *χ* ^2^ tests, as appropriate. The number of person-years of follow-up was calculated from the baseline date to the diagnosis of outcomes, death, loss to follow-up, or December 31, 2014, whichever occurred first [27]. The primary outcome was incident IS.

Crude incidence rate (CIR) for IS was calculated by the number of new incidents of diagnosed IS divided by the number of observed person-years. SIR and its 95% confidence interval (CI) were calculated as the ratio of the observed to the expected number of newly diagnosed cancer cases with the Poisson regression model, in which sex (male or female) and education level (illiteracy, below college, or above college) were entered as categorical variable and factors such as age, FBG, TC, HDL-C, LDL-C, and triglyceride (TG) as continuous variables. The methodology followed the details of our published article [28]. Linear trends were tested by the Cochran-Armitage test for categorical variables and means test for continuous variables. *P* values less than 0.05 were considered statistically significant. All statistical analyses were conducted by SAS version 9.4 (SAS Institute, Cary, NC, USA).

## 3. Results

### 3.1. Baseline Characteristics

A total of 22,795 subjects completed the follow-up, while 2335 subjects were lost to follow-up (Supplementary Table S1 and Supplementary Table S2). Baseline characteristics of the newly diagnosed T2D subjects stratified by BMI categories are shown in Table 1. Those who had normal BMI were older than those who were overweight and obese. Baseline characteristics stratified by height quartiles are presented in Table 2. Compared to taller counterparts, subjects who were shorter had higher FBG and HbA_1c_ levels in both males and females.

A total of 1268 newly diagnosed IS cases with 149,675 person-years were identified during the follow-up. The baseline characteristics of the IS cases are presented in Supplementary Table S3. For males, the average height and BMI were 168.23 ± 9.88 cm and 22.68 ± 5.83 kg/m^2^, respectively; for females, the average height and BMI were 156.12 ± 10.54 cm and 23.01 ± 4.21 kg/m^2^, respectively.

### 3.2. SIRs of IS among Incidents of T2D Subjects by Different Age Groups

**Figure 1:**
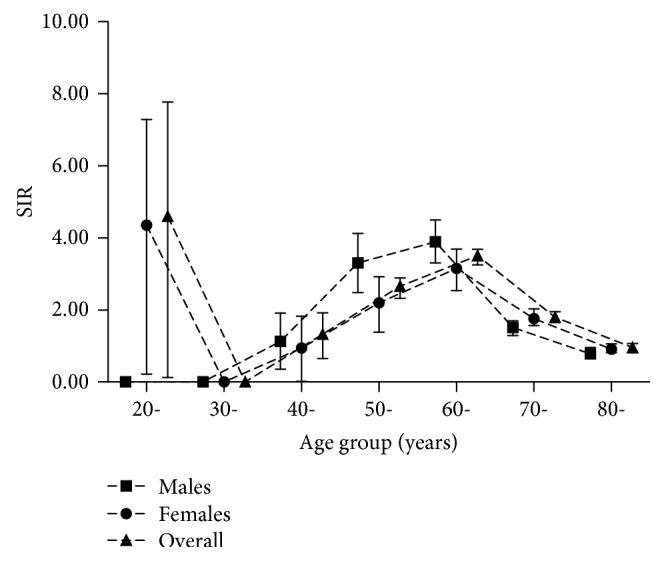
Standardized incidence ratios (SIRs) of ischemic stroke among incidents of type 2 diabetes subjects according to different age groups, adjusted for age, fasting blood glucose, total cholesterol, high-density lipoprotein cholesterol, low-density lipoprotein cholesterol, triglyceride, and education level.

SIRs of IS stratified by age are presented in Figure 1. Compared to the general population of Ningbo, SIRs increased with age until 60 years old. The 60+ age group had the highest risk for both males and females, with SIRs being 3.89 (95% CI 3.31–4.50) and 3.15 (95% CI 2.54–3.69), respectively, after adjusted for FBG, TC, HDL-C, LDL-C, TG, and education level. With the same adjustment, the overall SIRs for males and females were 1.41 (95% CI 1.29–1.53) and 1.45 (95% CI 1.32–1.57), respectively.

### 3.3. SIRs of IS among Incidents of T2D Subjects by Different BMI Categories

**Figure 2:**
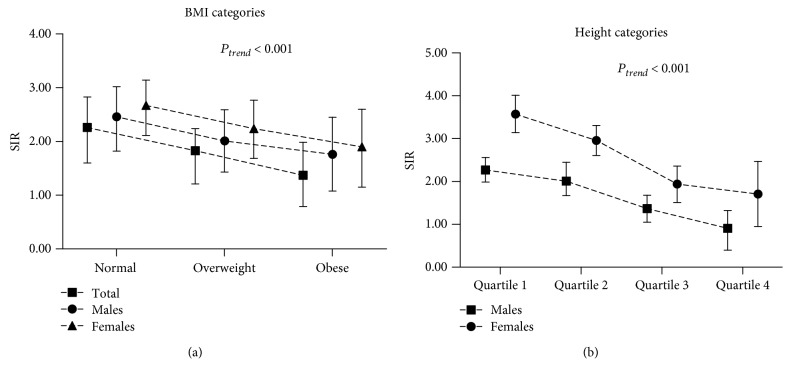
Standardized incidence ratios (SIRs) of ischemic stroke among incident type 2 diabetes subjects according to body mass index (BMI) categories and height quartiles, adjusted for age, fasting blood glucose, total cholesterol, high-density lipoprotein cholesterol, low-density lipoprotein cholesterol, triglyceride, and education level.

The overall SIRs in overall subjects were 2.56 (95% CI 1.90–3.13), 2.13 (95% CI 1.90–3.13), and 1.87 (95% CI 1.29–2.43) in normal BMI, overweight, and obese groups, respectively, after adjusted for age, FBG, TC, HDL-C, LDL-C, TG, and education level (*P*_trend_ < 0.01) (Figure 2). The SIR was 0.948 (95% CI 0.903–0.999) for each 1 kg/m^2^ increment in BMI.  Figure 2(a) shows that normal BMI subjects had higher SIRs than overweight and obese subjects for both males and females. With the same adjustment, the SIRs in males were 2.46 (95% CI 1.82–3.02), 2.01 (95% CI 1.43–2.59), and 1.76 (95% CI 1.08–2.45) in normal BMI, overweight, and obese groups (*P*_trend_ < 0.01), respectively. Each 1 kg/m^2^ increment in BMI was associated with 8% lower risk (SIR 0.922, 95% CI 0.877–0.970) of IS. The SIRs in females were 2.67 (95% CI 2.11–3.14), 2.24 (95% CI 1.68–2.77), and 1.90 (95% CI 1.15–2.60) in normal BMI, overweight, and obese groups (*P*_trend_ < 0.01), respectively. Each 1 kg/m^2^ increment in BMI was associated with 3% lower risk (0.977, 95% CI 0.933–1.022) of IS. Sensitivity analysis also showed similar results (Supplementary Figure S1).

### 3.4. SIRs of IS among Incidents of T2D Subjects by Different Height Quartiles

Figure 2 shows that each 1 cm increment in height had 7%, 8%, and 15% lower risk of IS in total subjects, males, and females, respectively. With the adjustment for age, FBG, TC, HDL-C, LDL-C, TG, and education level, the SIRs of IS stratified by different height quartiles are illustrated in Figure 2(b). For males, the SIRs for quartile 1 (≤160 cm), quartile 2 (161–165 cm), quartile 3 (166–170 cm), and quartile 4 (≥171 cm) were 2.27 (95% CI 1.99–2.56), 2.01 (95% CI 1.67–2.45), 1.37 (95% CI 1.05–1.68), and 0.91 (95% CI 0.40–1.32), respectively (*P*_trend_ < 0.01). For females, the SIRs were 3.57 (95% CI 3.14–4.01), 2.96 (95% CI 2.61–3.31), 1.94 (95% CI 1.51–2.36), and 1.71 (95% CI 0.95–2.47) for quartile 1 (≤155 cm), quartile 2 (156–160 cm), quartile 3 (161–165 cm), and quartile 4 (≥166 cm), respectively (*P*_trend_ < 0.01).

## 4. Discussion

This population-based prospective study suggested that newly diagnosed T2D had higher IS risk for all BMI categories and height quartiles. Compared to the subjects that were overweight and obese, newly diagnosed T2D subjects with normal BMI experienced higher risk of IS in the total, male, and female subjects.

Consistent with our study, Li et al. found that each 1 kg/m^2^ increase in BMI was associated with 1.7% lower risk of IS among 29,554 incidents in Americans with T2D [16]. Eeg-Olofsson et al. also reported that a 5 kg/m^2^ increase in BMI was related to increased risk of stroke among 13,087 incidents in T2D subjects [29]. In our study, subjects with normal BMI were older than the overweight and obese counterparts, and increased age is an established risk factor for stroke [30]. However, a recent study demonstrated that obesity was significantly associated with increased incident risk of cardiovascular (CVD) and mortality from CVD in 10 prospective cohort studies among North Americans [31]. And the China Kadoorie Biobank (CKB) study found that every 5 kg/m^2^ higher BMI was associated with 30% increased risk of IS among over 0.5 million normal Chinese adults [32]. The discrepancy among different studies may be due to the differences in study design, sample size, duration of follow-up, and age at recruitment. Notably, the inverse association was attenuated in both males and females, and female T2D subjects had higher IS risk than male counterparts. The levels of LDL-C were higher in females than in males in our study, and females had congenitally smaller-caliber coronary arteries than males, both of which are associated with increased risk of IS [33, 34].

In our study, we observed an inversed association between increased height and IS risk in both males and females. Compared to subjects with shorter heights, subjects with taller heights were younger than their counterparts, and age is a well-known risk factor for IS [35]. In accordance with our findings, a Japanese cohort observed that taller subjects were younger than shorter subjects, and each 5 cm increase in height was significantly inversely related to stroke risk [18]. Other prospective cohort studies also observed similar associations [17, 36, 37]. Taller subjects had a lower risk of stroke due to stronger pulmonary function and thicker coronary vessel diameters, which reduced the risk of vessel occlusion, thus contributing to decreased risk of IS [38].

Socioeconomic factors, such as education [33], income [6], and wealth [39], are important confounding factors for height, because people in higher socioeconomic classes tend to be taller than people in lower socioeconomic classes. Similar associations were reported in the association between BMI, education, and T2D [40]. Also, several biochemical indexes, such as FBG, LDL-C, and LDL-C, are also confounding factors for BMI or height, and most previous studies adjusted those indexes in the statistical models [16, 41, 42]. Therefore, we also adjusted those variables in our models.

To the best of our knowledge, we specifically estimated the incident of IS among newly diagnosed T2D subjects based on different BMI groups and height quartiles in the Chinese population. The larger sample size and longer duration of follow-up, and the inclusion of only newly diagnosed T2D subjects without preexisting cardiovascular disease at baseline, minimized the effect of T2D duration and preexisting diseases on IS risk. Moreover, we excluded subjects who were underweight, as lower BMI might indicate an underlying illness and susceptibility to IS [11].

Nevertheless, the following limitations should be acknowledged. Our data were obtained from the CDSS of Ningbo, and some important confounding factors were not available, such as smoking, alcohol consumption, and physical activity, which limited our further analysis. Besides, BMI was employed as the measure of adiposity, which does not reflect the overall fat distribution. Studies reported that waist circumference or waist-to-height ratio (WHR) might provide additional information beyond BMI for both incidence and mortality risk among middle-aged adults [43, 44]. And the generalizability of our findings is limited to newly diagnosed T2D subjects in Ningbo. Last, we could not stratify age into groups according to the life course (young, middle age, and older adulthood).

## 5. Conclusion

In conclusion, compared to the general population of Ningbo, T2D subjects had higher incident risk of IS. Newly diagnosed T2D subjects with normal BMI had a higher risk of IS compared to those who were overweight and obese, and increased height was related to decreased IS risk.

## Figures and Tables

**Table 1:** Baseline characteristics of T2D subjects according to different BMI categories.

Variables	18.5–24.0 kg/m^2^			*P*	24.0–28.0 kg/m^2^			*P*	≥28.0 kg/m^2^			*P*
	Total	Males	Females		Total	Males	Females		Total	Males	Females	
Numbers (%)	12,318 (54.04%)	5913 (48.00%)	6405 (52.00%)	<0.001	8790 (38.56%)	4131 (46.91%)	4659 (53.09%)	<0.001	1687 (7.40%)	833 (49.38%)	854 (50.62%)	<0.001
Age (year)	65.71 ± 12.85	64.33 ± 11.91	65.86 ± 13.09	<0.001	64.03 ± 12.38	62.95 ± 12.47	64.94 ± 1183	<0.001	60.79 ± 14.10	58.42 ± 13.89	61.27 ± 15.10	<0.001
BMI (kg/m^2^)	21.25 ± 3.16	21.77 ± 2.99	20.93 ± 3.05	<0.001	25.46 ± 1.10	26.88 ± 2.30	24.39 ± 1.76	<0.001	30.58 ± 8.38	32.46 ± 6.59	28.93 ± 8.16	<0.001
Education level (%)				<0.001				<0.001				<0.001
Illiteracy	4495 (36.49%)	1642 (36.53%)	2853 (63.47%)		1895 (21.59%)	863 (45.56%)	1032 (55.44%)		308 (18.26%)	132 (42.88%)	176 (57.12%)	
Below college	6608 (53.65%)	3475 (52.59%)	3133 (47.41%)		6227 (70.84%)	2891 (46.42%)	3336 (53.58%)		1219 (72.56%)	609 (49.95%)	610 (50.05%)	
Above college	1245 (9.86%)	796 (63.94%)	449 (36.06%)		668 (7.57%)	449 (67.22%)	219 (33.78%)		160 (9.18%)	92 (57.50%)	68 (42.50%)	
FBG (mmol/L)	9.19 ± 2.19	9.31 ± 1.67	9.04 ± 2.25	0.883	9.33 ± 2.99	9.42 ± 2.87	9.28 ± 3.31	0.562	9.76 ± 3.10	9.91 ± 3.48	9.65 ± 2.90	0.681
OGTT (mmol/L)	12.36 ± 3.96	13.24 ± 4.23	12.08 ± 3.77	0.322	12.67 ± 4.23	12.71 ± 3.94	12.59 ± 4.74	0.677	13.11 ± 4.03	13.37 ± 3.59	13.20 ± 2.99	0.203
TC (mmol/L)	4.76 ± 1.22	4.62 ± 1.17	4.84 ± 1.52	0.738	5.03 ± 1.59	4.95 ± 1.63	5.13 ± 1.44	0.481	6.02 ± 2.10	5.89 ± 2.47	6.28 ± 2.19	0.889
HDL-C (mmol/L)	1.41 ± 1.09	1.35 ± 1.17	1.46 ± 1.08	0.923	1.77 ± 1.27	1.74 ± 1.52	1.83 ± 1.19	0.717	1.15 ± 1.04	1.10 ± 1.02	1.22 ± 1.07	0.552
LDL-C (mmol/L)	2.82 ± 1.01	2.74 ± 1.37	2.88 ± 1.02	0.153	3.07 ± 1.56	2.95 ± 1.79	3.12 ± 1.56	0.244	3.21 ± 1.79	3.14 ± 1.86	3.27 ± 1.75	0.396
TG (mmol/L)	2.13 ± 1.89	2.12 ± 2.01	2.13 ± 1.87	<0.001	2.39 ± 1.77	2.35 ± 1.70	2.42 ± 1.29	<0.001	2.80 ± 2.41	2.76 ± 2.33	2.83 ± 1.92	<0.001
HbA_1c_ (%)	8.66 ± 2.51	8.81 ± 2.69	8.58 ± 2.11	<0.001	8.71 ± 2.13	8.89 ± 2.56	8.45 ± 2.03	<0.001	8.75 ± 3.08	9.11 ± 4.10	8.33 ± 3.65	<0.001

Data are presented as mean ± SD or number (percentage). FBG: fasting blood glucose; OGTT: oral glucose tolerance test; TC: total cholesterol; HDL-C: high-density lipoprotein cholesterol; LDL-C: low-density lipoprotein cholesterol; TG: triglyceride; HbA_1c_: glycosylated hemoglobin.

**Table 2:** Baseline characteristics of T2D subjects according to height quartiles.

Variables	Males				*P*	Females				*P*
	≤161 cm	161–165 cm	166–170 cm	≥171 cm		≤155 cm	156–160 cm	161–165 cm	≥166 cm	
Numbers (%)	1126 (11.78%)	1638 (17.13%)	3868 (40.45%)	2930 (30.64%)		3263 (24.66)	6085 (45.98%)	3093 (23.37%)	792 (5.99%)	
Age (year)	68.04 (13.57)	66.31 (12.22)	64.53 (12.50)	62.29 (12.37)	<0.001	68.79 (13.14)	65.13 (12.45)	62.37 (13.00)	61.45 (13.02)	<0.001
BMI (kg/m^2^)	22.09 (7.64)	23.32 (3.37)	23.08 (3.07)	23.29 (3.14)	0.059	22.95 (7.09)	23.11 (3.13)	23.20 (3.30)	22.90 (3.46)	0.0601
Education level (%)					<0.001					<0.001
Illiteracy	266 (23.64%)	262 (16.00%)	610 (15.77%)	408 (13.92%)		1250 (38.31%)	1428 (23.47%)	744 (24.05%)	194 (24.49%)	
Below college	768 (68.21%)	1264 (77.17%)	2887 (74.64%)	2099 (71.64%)		1783 (54.64%)	4136 (67.97%)	2123 (68.46%)	543 (68.56%)	
Above college	92 (8.15%)	112 (6.83%)	371 (9.59%)	423 (14.44%)		230 (7.05%)	521 (8.56%)	226 (7.49%)	55 (6.95%)	
FBG (mmol/L)	9.93 (3.20)	9.88 (3.14)	9.82 (4.19)	9.77 (4.23)	0.004	9.32 (4.14)	9.21 (3.12)	9.00 (2.39)	8.98 (3.29)	0.002
OGTT (mmol/L)	14.62 (4.10)	14.33 (3.93)	13.92 (5.01)	13.79 (5.12)	0.018	12.06 (4.61)	12.02 (4.11)	11.96 (3.49)	11.90 (4.17)	0.019
TC (mmol/L)	5.11 (1.40)	5.04 (1.33)	4.93 (1.89)	4.88 (1.92)	0.351	5.29 (1.93)	5.27 (1.41)	5.23 (1.21)	5.19 (1.42)	0.360
HDL-C (mmol/L)	1.26 (0.79)	1.25 (0.66)	1.22 (0.90)	1.20 (1.05)	0.434	1.55 (1.43)	1.49 (1.26)	1.47 (0.87)	1.41 (1.27)	0.470
LDL-C (mmol/L)	2.91 (1.23)	2.87 (1.02)	2.79 (1.51)	2.77 (1.45)	0.381	3.09 (1.58)	3.00 (1.08)	2.98 (1.02)	2.93 (1.09)	0.384
TG (mmol/L)	2.49 (1.80)	2.44 (1.68)	2.30 (1.99)	2.21 (2.07)	0.395	2.49 (2.21)	2.42 (1.98)	2.40 (0.99)	2.39 (2.06)	0.402
HbA_1c_ (%)	8.75 (3.04)	8.72 (2.88)	8.62 (3.41)	8.63 (3.22)	0.301	8.33 (3.11)	8.27 (2.06)	8.26 (1.77)	8.21 (2.29)	0.311

Data are presented as mean (SD) or number (percentage). FBG: fasting blood glucose; OGTT: oral glucose tolerance test; TC: total cholesterol; HDL-C: high-density lipoprotein cholesterol; LDL-C: low-density lipoprotein cholesterol; TG: triglyceride; HbA_1c_: glycosylated hemoglobin.

## Data Availability

Previously reported data were used to support this study and are available at [doi:10.1016/j.canep.2018.02.006]. These prior studies (and datasets) are cited at relevant places within the text as references [28].

## Supplementary Materials

Supplementary Materials Supplementary Table S1: baseline characteristics of 22,795 T2D subjects. Supplementary Table S2: baseline characteristics of the lost to follow-up subjects. Supplementary Table S3: baseline characteristics of 1268 T2D subjects with ischemic stroke. Supplementary Figure S1: SIR of IS among T2D subjects according to BMI categories.
