# Supplementary material for: Does Body Mass Index and Height Influence the Incident Risk of Ischemic Stroke in Newly Diagnosed Type 2 Diabetes Subjects?
Source: J Diabetes Res. 2019 Jan 22;2019:2591709. doi: 10.1155/2019/2591709 (PMC6362488; doi:10.1155/2019/2591709)
Supplement: Supplementary Materials — Supplementary Table S1: baseline characteristics of 22,795 T2D subjects. Supplementary Table S2: baseline characteristics of the lost to follow-up subjects. Supplementary Table S3: baseline characteristics of 1268 T2D subjects with ischemic stroke. Supplementary Figure S1: SIR of IS among T2D subjects according to BMI categories. [file 2591709.f1.pdf]

Supplementary Materials

Supplementary Table S1. Baseline Characteristics of 22,795 T2D subjects

Supplementary Table S2. Baseline characteristics of the lost follow-up subjects

Supplementary Table S3. Baseline Characteristics of 1,268 T2D subjects with ischemic stroke

Supplementary Figure S1. SIR of IS among T2D subjects according to BMI categories

Supplementary Table S1

Baseline Characteristics of 22,795 incident T2D subjects

Variables	Total	Males	Females	*P*-value
Sex (%)	22795	9562(41.9%)	13233(59.1%)	0.23
Age(year)	65.17 (12.82)	64.59 (12.51)	65.59 (13.02)	<0.01
Height(cm)	162.43 (11.98)	168.74 (11.09)	157.82 (10.40)	<0.01
BMI(kg/m^2)	23.14 (4.39)	23.16 (3.88)	23.12 (4.72)	0.55
Education level				<0.01
illiteracy	428(33.8%)	133(21.9%)	295(44.6%)	
below college	774(61.0%)	443(73.1%)	331(50.0%)	
above college	66 (5.2%)	30 (5.0%)	36 (5.4%)	
FBG(mmol/l)	9.46 (3.39)	9.89 (3.70)	9.11 (3.09)	0.02
OGTT(mmol/l)	12.87 (4.28)	14.46 (4.33)	12.01 (4.00)	0.03
TC(mmol/l)	5.16 (1.35)	5.07 (1.42)	5.25 (1.25)	0.65
HDL-C(mmol/l)	1.38 (1.10)	1.24 (0.89)	1.53 (1.26)	0.04
LDL-C(mmol/l)	2.93 (1.06)	2.88 (1.02)	2.99 (1.10)	0.59
TG(mmol/l)	2.41 (2.33)	2.41 (2.73)	2.41 (1.82)	0.82
HbA_{1c} (%)	8.70 (2.42)	9.07 (2.35)	8.24 (2.47)	0.02

Data are presented as mean (SD) or number (percentage).FBG: fasting blood glucose; OGTT: oral glucose tolerance test; TC: total cholesterol; HDL-C: high density lipoprotein cholesterol; LDL-C: low-density lipoprotein cholesterol;TG: triglyceride; HbA_{1c}: glycosylated hemoglobin.

Supplementary Table S2

Baseline characteristics of the lost follow-up subjects

Variables	Total	Males	Females	*P*-value
Sex (%)	2335	1046(44.8%)	1289 (55.2%)	0.47
Age(year)	64.99±12.56	64.12±12.39	65.38±12.77	0.23
Height(cm)	163.12±12.27	167.51±12.63	158.13±11.33	0.08
BMI(kg/m^2)	23.74±4.28	23.97±4.01	23.27±4.45	0.55
Education level				<0.01
Illiteracy	7704 (33.8%)	2094 (21.9%)	5901 (44.6%)	
Below college	13904 (61.0%)	6989 (73.1%)	6616 (49.9%)	
Above college	1187 (5.2%)	479 (5.0%)	716 (5.5%)	
FBG(mmol/l)	9.53±3.88	9.92±3.41	9.09±3.77	0.36
OGTT(mmol/l)	12.65±4.58	14.12±4.09	12.25±4.68	0.02
TC(mmol/l)	5.20±1.28	5.08±1.38	5.29±1.35	0.65
HDL-C(mmol/l)	1.42±1.19	1.31±1.02	1.56±1.24	0.49
LDL-C(mmol/l)	2.88±1.05	2.79±1.05	2.96±1.14	0.16
TG(mmol/l)	2.48±2.15	2.39±2.26	2.51±1.64	0.53
HbA_{1c} (%)	8.87±2.63	9.15±2.48	8.39±2.27	<0.01

Data are presented as mean±SD or number (percentage).FBG: fasting blood glucose; OGTT: oral glucose tolerance test; TC: total cholesterol; HDL-C: high density lipoprotein cholesterol; LDL-C: low-density lipoprotein cholesterol; TG: triglyceride; HbA_{1c}: glycosylated hemoglobin.

Supplementary Table S3

Baseline Characteristics of 1,268 T2D subjects with ischemic stroke

Variables	Total	Males	Females	*P*-value
Sex(%)	1268	606(47.8%)	662(52.2%)	0.15
Age(year)	74.48±9.91	73.25±10.46	75.60±9.25	<0.01
Height(cm)	161.31±10.14	168.23±9.88	156.12±10.54	<0.01
BMI(kg/m^2)	24.33±3.21	25.12±3.08	23.77±4.12	0.32
Education level				<0.01
Illiteracy	393(31.0%)	163(26.8%)	230(34.7%)	
Below college	783(61.8%)	387(63.9%)	396(59.8%)	
Above college	92(7.2%)	56(9.3%)	36(5.5%)	
FBG(mmol/l)	9.62±3.81	9.92±4.11	9.27±3.23	<0.01
OGTT(mmol/l)	12.56±4.33	14.78±4.56	11.76±4.02	<0.01
TC(mmol/l)	5.30±1.61	5.39±1.77	5.23±1.42	0.54
HDL-C(mmol/l)	1.35±1.06	1.25±0.85	1.57±1.28	0.02
LDL-C(mmol/l)	2.90±1.05	2.86±1.01	2.97±1.12	0.41
TG(mmol/l)	2.52±2.41	2.44±2.71	2.68±1.96	0.77
HbA_{1c}(%)	8.67±4.39	8.98±4.31	8.31±4.49	0.04

Data are presented as mean ± SD or number (percentage); FBG: fasting blood glucose; OGTT: oral glucose tolerance test; TC: total cholesterol; HDL-C: high density lipoprotein cholesterol; LDL-C: low-density lipoprotein cholesterol; TG: triglyceride; HbA_{1c}: glycosylated hemoglobin.

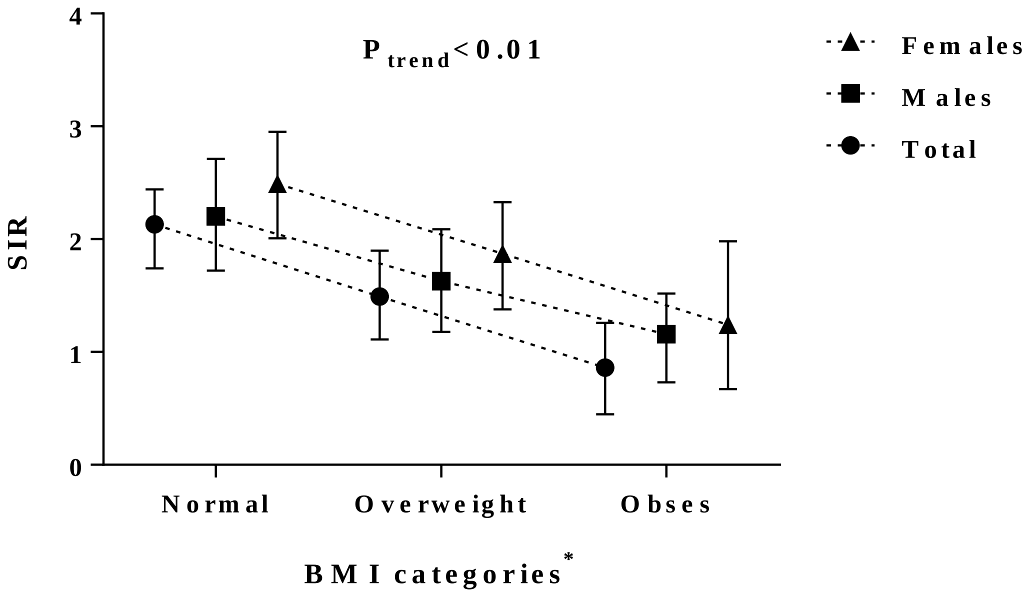

Supplementary Figure S1. SIR of IS among incident T2D subjects according to BMI categories**.**SIR, standardized incidence ratio; 95% CI, 95% confidence interval; IS, ischemic stroke.*According to WHO criteria:normal weight, 18.5-24.9 kg/m^2; overweight, 25.0-29.9 kg/m^2; obese, ≥30.0 kg/m^2 (due to excluded subjects those who were underweight [<18.5 kg/m^2], we did not calculate the SIR for underweight group).
